# Natural Antibodies as Rheostats for Susceptibility to Chronic Diseases in the Aged

**DOI:** 10.3389/fimmu.2016.00127

**Published:** 2016-04-07

**Authors:** Thomas L. Rothstein

**Affiliations:** ^1^Center for Oncology and Cell Biology, The Feinstein Institute for Medical Research, Manhasset, NY, USA

**Keywords:** human B cells, B-1 cells, natural antibody

## Abstract

Natural antibodies are spontaneously produced in the absence of infection or immunization, and are both anti-microbial and autoreactive. Autoreactive natural antibodies can bind noxious molecules, such as those involved in clinical situations of atherosclerosis (oxLDL), malignancy (NGcGM3), and neurodegeneration (amyloid, tau) and can affect the fate of their targets or the cells bearing them to maintain homeostasis. Clinically relevant natural antibodies have been shown to decline with advancing age in those few situations where measurements have been made. Consistent with this, human B-1 cells that are thought to be responsible for generating natural antibodies also decline with advancing age. These findings together suggest that an age-related decline in amount or efficacy of homeostatic natural antibodies is associated with relative loss of protection against molecules involved in several diseases whose incidence rises in the older age population, and that those individuals experiencing greatest loss are at greatest risk. In this view, natural antibodies act as rheostats for susceptibility to several age-related diseases. These considerations suggest that administration of natural antibodies, or of factors that maintain B-1 cells and/or enhance production of natural antibodies by B-1 cells, may serve to counteract the onset or progression of age-related chronic illness.

## Introduction

Natural antibody represents immunoglobulin that is spontaneously and constitutively secreted in the absence of infection or immunization. Natural antibody is present in animals and humans, and is thought to comprise the bulk of resting IgM, along with portions of isotype-switched IgA and IgG. Natural antibody differs from adaptive antibody in many ways, importantly including repertoire and function. Much natural antibody is anti-microbial and forms a preexisting shield against infection that provides a primary layer of protection during the lag period required for germinal center formation and adaptive antibody production (1–5). Natural antibody also tends to be autoreactive (6–9) and performs a second beneficial function in housekeeping and homeostatic activity that speeds elimination of dying cell debris and noxious molecular species (4, 10–17). In this way, potentially inflammatory and/or toxic agents are removed before direct tissue injury can occur.

Natural antibody is generated for the most part by a relatively small but unique subpopulation of B cells termed B-1 cells, first recognized in 1982, that is developmentally distinct (18–20). The origin and function of B-1 cells have been most extensively studied in mice, where B-1 cells are readily identified by a clear set of phenotypic markers (B220loCD5^+^CD23^−^CD43^+^IgMhiIgDlo). For some time, the status of human B-1 cells has been uncertain, and the existence of human B-1 cells has been debated. However, a new phenotypic profile for B-1 cells in human peripheral blood was recently reported (CD19^+^CD20^+^CD27^+^CD38modCD43^+^ CD70^−^) (21–23) and, despite some controversy (24–27), this profile has gained acceptance and has been utilized by a number of investigators in translational studies of specific disease states (25, 26, 28–31).

The natural antibodies produced by B-1 cells differ in sequence from adaptive antibodies produced by conventional B (B-2) cells, which in turn dictates repertoire and function. Mouse B-1 cell antibodies are more germ line-like in comparison to mouse B-2 cell antibodies by virtue of containing little or no somatic hypermutation and much reduced, or non-existent, N-region addition (15, 32–34) both of which affect CDR3 domains that are major contributors to antigen binding. The lack of N-addition appears to derive from the absence of terminal deoxynucleotidyl transferase (TdT) during mouse hematopoietic development early in life when the bulk of B-1 cells are generated (35). However, human B-1 cell antibodies often contain N-addition, which likely reflects the presence of TdT throughout ontogeny in *Homo sapiens* (35). Like mouse B-1 cell antibodies, human B-1 cell antibodies contain little or no somatic hypermutation early in life (21), but acquire somatic mutation as time goes on, although some difference in this measure between B-1 and B-2 cell antibodies continues into adulthood (23). Because B-1 cell antibodies tend to reflect sequences delineated in the genome with little alteration, especially in mice, it has been suggested that the B-1 cell repertoire is “tuned” over evolutionary time, obeying Darwinian precepts such that sequences functioning to promote survival are retained (10). In this view, B-1 cell antibodies represent the best functioning antibodies for the roles that they fulfill.

## Human Natural Antibodies Recognize Molecules Associated with Diseases of Aging

Human natural antibodies directed against a variety of molecules with clinical significance have been identified. Three specific disease areas are illustrative, and these are three of the most common, distressing, and burdensome diseases associated with aging. (1) *Atherosclerosis* : healthy individuals commonly express IgM antibodies that bind oxidized low-density lipoproteins (oxLDL) (36). Oxidized LDLs arise from non-enzymatic processes, accumulate within vessel walls, and contribute to plaque formation and inflammation that together drive the disease process of atherosclerosis, resulting in cardiovascular events that can be lethal (37). One type of anti-oxLDL natural antibody binds an oxidized form of the major lipoprotein, apolipoprotein B100 (38–40). (2) *Malignancy*: healthy individuals commonly express antibodies that bind *N*-glycolylneuraminyl-lactosylceramide (NGcGM3) (41). NGcGM3 is not thought to be produced in human tissues due to an inactivating insertional mutation of cytidine monophosphate-*N*-acetylneuraminic acid hydroxylase (CMAH) that occurred evolutionarily after divergence of humankind from great apes, about 2.8 million years ago (42, 43). However, NGcGM3 is present in humans, presumably acquired exogenously by dietary intake, and for reasons that are as yet unclear is concentrated many fold in the membranes of some tumors, prominently including the malignant cells of lung cancer (44). (3) *Neurodegeneration*: healthy individuals commonly express antibodies that bind amyloid and tau proteins (45–48). Abnormal plaques (amyloid) and tangles (tau) of these proteins have been implicated in the pathogenesis of Alzheimer’s Disease, in which protein aggregates result in neuronal dysfunction, and enhanced phosphorylation may play a role in this abnormal protein behavior and disease pathogenesis (49).

## Disease-Associated Natural Antibodies are Functional

Natural antibodies directed against antigens associated with these three classes of disease appear to be functional. (1) In mice, a number of adoptive transfer experiments with *Apoe^−/−^* recipients have led to the generally accepted paradigm that B-1 cells and the IgM antibodies they produce are atheroprotective, whereas B-2 cells and the IgG antibodies they produce are atherogenic (50, 51). Less invasive studies have been carried out with people, and it has been shown that human IgM anti-oxLDL is inversely correlated with cardiovascular and carotid disease (12, 38, 39, 52–54), whereas IgG has been found to be positively correlated with atherosclerosis (12, 52, 55–60) or not correlated at all with vessel pathology (40, 61–64). The mechanism appears to involve inhibition of oxLDL uptake by macrophages (65, 66). In a recent study, human serum antibodies directed against a methylglyoxal (MGO) modified apolipoprotein B100 peptide were examined. The levels of IgM antibodies in healthy individuals aged 63–68 were found to be inversely correlated with cardiovascular events occurring during the subsequent 15 years; in contrast, the levels of IgG antibodies were not correlated with subsequent cardiovascular events (67). Thus, in both mouse and human, natural IgM antibodies against oxLDL, appear to counteract the development of atherosclerosis. (2) Human natural anti-NGcGM3 antibodies have been shown to specifically bind and eliminate malignant cells bearing NGcGM3. This tumor cell destruction by anti-NGcGM3 antibodies occurs through both a complement-dependent mechanism and an oncosis-like, complement-independent mechanism (41, 68, 69). Somewhat akin to the correlative results noted above with respect to MGO-modified apoB100 peptide, patients with lung cancer lack or have very low levels of anti-NGcGM3 antibodies (41). Separately, an anti-idiotypic antibody vaccine (racotumomab) that displays the “internal image” of NGcGM3 has been developed to stimulate production of anti-NGcGM3 antibodies (69–71). In a recent clinical trial for maintenance treatment after first line chemotherapy in non-small cell lung cancer patients, racotumomab significantly prolonged overall survival and progression free survival, and those patients experiencing the greatest antibody response had the best outcomes (69, 72). Thus, natural and elicited cytotoxic antibodies against NGcGM3 appear to protect against the onset and/or ameliorate the course of lung cancer. (3) Human natural antibodies against amyloid and tau have been proposed as agents that might oppose and/or treat Alzheimer’s neurodegeneration. As with the inverse correlation between serum levels of natural antibodies and the disease states of atherosclerosis and malignancy discussed above, natural anti-amyloid antibodies have been shown to be relatively diminished in patients with Alzheimer’s Disease (46, 73–75). These natural anti-amyloid antibodies have been shown to diminish the burden of aggregated proteins and improve cell viability *in vitro* (45, 46, 76, 77). In animal studies, passive administration of antibodies against amyloid and tau has in each case depleted abnormal proteins from the brain and improved pathology and/or behavioral parameters (76, 78–81). In recent clinical trials, passive administration of monoclonal antibodies against amyloid protein failed to produce improvement in cognition or function (82–85). This failure of clinical improvement in anti-amyloid trials to date, despite preclinical data showing diminished protein aggregation, remains unexplained, but may suggest the utility of alternative anti-tau treatment. Regardless, these results indicate that circulating antibodies can affect aggregation and alter deposits of abnormal, pathological amyloid and tau proteins.

## Clinically Relevant Natural Antibodies are Diminished or Less Effective with Increasing Age and Disease

In each of the three clinical entities discussed above, natural antibodies that recognize disease-associated epitopes are diminished in affected patients. There are several potential explanations for these inverse correlations, among which is the possibility that the absence of homeostatic antibodies increases the risk of developing disease. This is perhaps most directly suggested by the prospective study of natural antibodies that recognize modified apoB100 and the associated subsequent risk of cardiovascular events, discussed above. These diseases of atherosclerosis, malignancy, and neurodegeneration are all more common with increasing age. If natural antibodies are involved in opposing disease pathogenesis and/or disease progression, it would be expected that levels of disease-related natural antibodies would be found to be diminished with advancing age. In fact, an age-related decline has been documented for natural antibodies directed against NGcGM3 (41), and for natural antibodies directed against amyloid (46). Thus far, the relationship between natural antibodies against oxidized apoB100 and age has not been examined.

## B-1 Cells Generate Homeostatic Antibodies

In mice, natural antibodies are predominantly, if not exclusively, generated by B-1 cells. The recent phenotypic identification of human B-1 cells raises the question of whether this population is responsible for producing human natural antibodies, especially those related to disease. This has been evaluated for atherosclerosis-predictive/-protective IgM antibodies against MGO-modified apoB100. Among human B-1 cell, memory B cell, preplasmablast and plasmablast culture supernantants, natural IgM anti-MGO-apoB100 antibodies were generated predominantly by human B-1 cells (67). Similarly, B-1 cells are responsible for producing natural anti-NGcGM3 antibodies in mice (44). However, human B-1 cells have not yet been tested for production of antibodies neither against NGcGM3 nor against amyloid and tau.

## Age-Related Changes in Natural Antibodies Likely Relate to Age-Related Changes in B-1 Cells

To the extent that human B-1 cells are the origin of disease-related homeostatic natural antibodies, then a change in B-1 cells may underlie the decline that occurs with advancing age. To address this possibility, B-1 cell and other B cell populations were enumerated in peripheral blood of healthy adult volunteers over a wide age range. This study showed an age-related decline in B-1 cells (21). Other B cell populations did not change with age. Thus, B-1 cell numbers are age-sensitive. Although some investigators have reported an age-related decline in memory B cells, others have not (86–89), but B-1 cells were not differentiated from CD27^+^ memory B cells in earlier studies.

Beyond numbers, there is some evidence in mouse studies that the B-1 cell repertoire changes with age (90). In a careful study involving deep sequencing, Ghosn et al. showed that selection operates on the B-1 cell repertoire as mice mature (91). Consistent with this, the avidity of natural anti-amyloid antibodies is diminished in patients with Alzheimer’s Disease as compared to healthy controls (75). Thus, as a result of declining B-1 cell numbers, or a change in B-1 cell repertoire, or both, natural antibody deteriorates, which appears to be accompanied by a loss of the protection, especially homeostatic protection, that natural antibody affords.

## The Rheostat Hypothesis for B-1 Cell Natural Antibodies

Weaving these different strands of evidence together, there is reason to hypothesize, as a general paradigm, that: (1) an age-related decline in amount and/or efficacy of homeostatic natural antibodies is in turn associated with relative loss of protection against molecules involved in several diseases whose incidence rises in the older age population; and, (2) those individuals experiencing the greatest loss in amount and/or efficacy of homeostatic natural antibodies are at greatest risk. In this view, natural antibodies act as rheostats for susceptibility to several age-related diseases that are associated with accumulation of noxious molecules or involve unique molecular targets, or both. Extrapolation from this point suggests the possibility that administration of disease-opposing natural antibodies or of factors that maintain B-1 cells and/or enhance production of disease-opposing natural antibodies by B-1 cells could serve to counteract the onset or progression of age-related chronic illness.

## Why are Natural Antibodies Relevant to Diseases of the Elderly Present Beyond the Age of Reproduction?

According to Darwinian principles, there is no advantage to counteracting diseases whose onset occurs after reproductive age. In this sense, then, results in both the mouse and human systems raise the question of why natural antibodies that protect against age-associated diseases are retained in evolution. This likely results from polyreactivity of B-1 cell natural antibodies, and antigenic mimicry of B-1 cell natural antibody targets, as illustrated graphically by mouse T15/E06 (11, 92). T15 is a completely germ-line antibody that arises in many mouse strains, first identified by its recognition of phosphorylcholine, an antigenic determinant found on pneumococci and other microbes. E06 is a completely germ-line antibody identified by its binding to oxidized LDL. T15 and E06 are, in fact, one-and-the-same; they are identical antibodies that protect against pneumococcal infection and affect the disposition of oxLDL (93). Moreover, human antibodies with these kinds of specificities appear early in life. This is highlighted by reports that IgM anti-oxLDL antibodies are found in umbilical cord blood samples and in blood samples from preterm and full-term infants (94, 95). These antibodies block the uptake of oxLDL by macrophages and are often germ line in heavy chain sequence (95). So natural antibodies capable of influencing atherosclerosis later in life appear early in ontogeny and seem to exist by virtue of a combination of similarity between antigens on bacteria and oxidized lipids and polyreactivity of B-1 cell-derived natural antibodies. The same is likely true of other natural antibodies.

## Do Natural Antibodies Arise Spontaneously or are They Stimulated by Self-Antigens?

Mature B-1 cells secrete antibody spontaneously and constitutively, in the absence specific antigen engagement, which fails to generate typical signs of BCR signaling and activation in these cells (96). However, the BCR may play a role early on. Studies in mice indicate that B-1 cell development is enhanced by antigen engagement, the inverse of antigen-induced apoptosis in nascent B-2 cells (97, 98). The relevant antigens may be self-antigens, inasmuch as B-1 cell development is not disturbed in germ-free mice lacking foreign antigens (91). NGcGM3 would appear to contradict this paradigm because it cannot be a self-antigen in the human species that lacks CMAH and is incapable of generating this ganglioside. However, the germ-line antibody, 4ac, which binds myelin oligodendrocyte glycoprotein (MOG), a central nervous system target for EAE (experimental allergic encephalomyelitis), is identical to the germ-line anti-NGcGM3 antibody, P3, and so cross-reactivity with self components may explain the existence of natural antibodies against NGcGM3 (99). Further, potential transfer of NGcGM3 across the placenta and in mother’s milk at early stages of fetal/neonatal development is unknown. Overall, the degree to which the B-1 cell repertoire is shaped by self-antigens as opposed to “foreign” antigens remains a question yet to be fully resolved. In light of the polyreactivity and antigen mimicry discussed above, the determinants of the B-1 cell repertoire are likely to have an important influence on the level of homeostatic protection provided by B-1 cell natural antibody and may be responsible, at least in part, for the variation in protective antibody noted among older individuals.

## Other Questions Remain

B-1 cells in mouse and human can isotype switch and secrete natural antibodies that are IgA and IgG as well as IgM (23, 100, 101). The degree to which this happens may be important in assessing the level of homeostatic protection afforded by natural antibodies. For example, IgM anti-oxLDL antibodies protect against atherosclerosis in mice and correlate with protection against cardiovascular events in humans, whereas IgG anti-oxLDL antibodies do not. However, it is unknown at present whether IgG anti-oxLDL antibodies originate from B-1 cells and whether, if they do, they can be as protective as IgM anti-oxLDL antibodies.

In addition, other B cell populations, such as marginal zone B cells and IgM memory B cells, have been proposed as contributors to the pool of natural antibodies (102–106). At present it is unknown to what extent, if any, protective homeostatic antibodies derive from these populations but this could be relevant to the extent that enhancement of homeostatic natural antibody producing B cell populations becomes a prophylactic or therapeutic maneuver in the future.

## Conclusion/Rheostat Redux

To summarize, it is proposed that many chronic diseases associated with aging, including atherosclerosis, cancer, and neurodegeneration, and possibly others, take place against a background of greater or lesser homeostatic protection provided by B-1 cell-derived natural antibodies that decline with advancing age due to a decrease in the B-1 cell population and/or an alteration in the B-1 cell repertoire. There is much to be learned regarding the development and function of B-1 cells and the nature of homeostatic natural antibodies, and changes that occur in both B-1 cells and natural antibodies with advancing age. As this information is acquired, it is proposed that the onset and/or course of several chronic diseases of aging might be favorably altered by therapies that maintain or enhance the native B-1 cell population and/or that replace or add exogenous natural antibodies.

## Author Contributions

TR conceived and wrote the perspective based on work from his laboratory and others over many years time.

## Conflict of Interest Statement

The author declares that the research was conducted in the absence of any commercial or financial relationships that could be construed as a potential conflict of interest.
